# Supplementary material for: Influence of Prolonged Visual Display Terminal Use on Physical and Mental Conditions among Health Care Workers at Tertiary Hospitals, Taiwan
Source: Int J Environ Res Public Health. 2022 Mar 22;19(7):3770. doi: 10.3390/ijerph19073770 (PMC8998003; doi:10.3390/ijerph19073770)
Supplement: Supplementary file 1 [file ijerph-19-03770-s001.zip › ijerph-1528441-supplementary.pdf]

Supplement Table S1 Demography and characteristics of personal habits and working characters among hospital employees

**Variables**	**Non-doctor/nurse (*n* = 945)**		**Doctor/nurse (*n* = 1868)**		***p* value**
Age, years	44.68 ± 11.20		35.29 ± 10.96		<0.001
Age (*n*, %)					<0.001
<30	128	13.54%	801	42.88%	
≧30	817	86.46%	1067	57.12%	
Women, *n* (%)	771	81.59%	1745	93.40%	<0.001
Education level, *n* (%)					<0.001
≦Senior high school	326	34.50%	45	2.41%	
College	499	52.80%	1698	90.90%	
≧Graduate School	120	12.70%	125	6.69%	
Seniority, n (%)					0.001
< 2 years	159	16.84%	370	19.81%	
2-4 years	164	17.37%	307	16.43%	
4-10 years	243	25.74%	566	30.30%	
> 10yeras	378	40.04%	625	33.46%	
Working hours/week, *n* (%)					<0.001
≦ 45 hrs	740	78.39%	1058	56.64%	
46-50 hrs	185	19.60%	609	32.60%	
51-59 hrs	14	1.48%	115	6.16%	
≧60 hrs	5	0.53%	86	4.60%	
Work style, *n* (%)					<0.001
Regular class	692	73.23%	753	40.31%	
Night shift	17	1.80%	116	6.21%	
Three shifts	236	24.97%	999	53.48%	
Smoking, *n* (%)	49	5.19%	54	2.89%	0.002
Drink, *n* (%)	64	6.77%	123	6.58%	0.85
Exercise frequency (hu/week), *n* (%)					<0.001
never	373	39.47%	959	51.34%	
1 time per week	276	29.21%	483	25.86%	
2-4 times per week	257	27.20%	380	20.34%	
≧5 times per week	39	4.13%	46	2.46%	

* Smoking status (current or past/never), alcohol consumption (0-1 drinks per week/≧2 drinks per week), exercise (≧30 mins/time)

Supplement Table S2 Prevalence of musculoskeletal and visual system among hospital employees

Variables	Non-doctor/nurse (*n* = 945)		Doctor/nurse (*n* = 1868)		*p* value
Musculoskeletal pain, *n* (%)					
Neck	514	54.39%	1244	66.60%	<0.001
Shoulders	629	66.56%	1413	75.64%	<0.001
Back	383	40.53%	784	41.97%	0.46
Elbows	260	27.51%	392	20.99%	<0.001
Lower back or waist	511	54.07%	1189	63.65%	<0.001
Wrists or hands	391	41.38%	702	37.58%	0.05
Hips or legs	210	22.22%	492	26.34%	0.02
Number of pain area	2.90 ± 1.81		3.05 ± 2.06		0.04
Eye discomfort, *n* (%)					0.02
Nerve	375	39.68%	641	34.31%	
Mild-moderate	432	45.71%	924	49.46%	
Severe	138	14.60%	303	16.22%	
Headache, *n* (%)					<0.001
Nerve	455	48.15%	707	37.85%	
Mild-moderate	450	47.62%	968	51.82%	
Severe	40	4.23%	193	10.33%	
Computer vision syndrome, *n* (%)					<0.001
Nerve	375	39.68%	641	34.31%	
Mild-moderate	187	19.79%	325	17.40%	
Severe	383	40.53%	902	48.29%	

Supplement Table S3 Prevalence of mental stress among hospital employees

Variables	Non-doctor/nurse (*n* = 945)			Doctor/nurse (*n* =1 868)			*p* value
BSRS-5 score (mean ± SD)	4.15	±	3.33	5.62	±	3.91	<0.001
BSRS-5, *n* (%)							<0.001
≦5	706		74.71%	1099		58.83%	
6-9	162		17.14%	456		24.41%	
10-14	68		7.20%	252		13.49%	
≧15	9		0.95%	61		3.27%	
Self-rated Health Status, n (%)							<0.001
Good	326		34.50%	501		26.82%	
Moderate	560		59.26%	1160		62.10%	
Poor	59		6.24%	207		11.08%	
Sleep duration, hours (mean, SD)	6.55		0.99	6.59		1.09	0.31
Self-assessment of sleep time							0.001
Good	416		44.02%	701		37.57%	
Poor	529		55.98%	1165		62.43%	
Burnout, *n* (%)	61		6.46%	122		6.53%	0.94
Maslach Burnout Inventory (MBI) (Mean, SD)							
Emotional exhaustion	40.29	±	19.19	49.98	±	19.80	<0.001
Personal accomplishment	52.14	±	11.05	57.42	±	11.08	<0.001
Depersonalization	29.48	±	18.57	38.14	±	19.35	<0.001
Job stress							<0.001
Mild (*n* = 1602)	682		72.17%	920		49.25%	
Moderate (*n* = 485)	140		14.81%	345		18.47%	
Sever (*n* = 726)	123		13.02%	603		32.28%	

n = numbers, SD = Standard deviation, BSRS = Brief Symptom Rating Scale, MBI = Maslach Burnout Inventory.
